# Secure and Decentralized Hybrid Multi-Face Recognition for IoT Applications

**DOI:** 10.3390/s25185880

**Published:** 2025-09-19

**Authors:** Erëza Abdullahu, Holger Wache, Marco Piangerelli

**Affiliations:** 1 Computer Science Division, School of Science and Technology, University of Camerino, Via Madonna Delle Carceri 7, 62032 Camerino, Italy; ereza.abdullahu@studenti.unicam.it; 2 School of Business, University of Applied Sciences and Arts Northwestern Switzerland FHNW, CH-4600 Olten, Switzerland; holger.wache@fhnw.ch; 3 Vici & C. S.p.A., Via Gutenberg 5, 47822 Santarcangelo di Romagna, Italy

**Keywords:** multi face-recognition, hybrid model, convolutional neural networks, Internet of Things, decentralization, edge AI, sensors, security

## Abstract

The proliferation of smart environments and Internet of Things (IoT) applications has intensified the demand for efficient, privacy-preserving multi-face recognition systems. Conventional centralized systems suffer from latency, scalability, and security vulnerabilities. This paper presents a practical hybrid multi-face recognition framework designed for decentralized IoT deployments. Our approach leverages a pre-trained Convolutional Neural Network (VGG16) for robust feature extraction and a Support Vector Machine (SVM) for lightweight classification, enabling real-time recognition on resource-constrained devices such as IoT cameras and Raspberry Pi boards. The purpose of this work is to demonstrate the feasibility and effectiveness of a lightweight hybrid system for decentralized multi-face recognition, specifically tailored to the constraints and requirements of IoT applications. The system is validated on a custom dataset of 20 subjects collected under varied lighting conditions and facial expressions, achieving an average accuracy exceeding 95% while simultaneously recognizing multiple faces. Experimental results demonstrate the system’s potential for real-world applications in surveillance, access control, and smart home environments. The proposed architecture minimizes computational load, reduces dependency on centralized servers, and enhances privacy, offering a promising step toward scalable edge AI solutions.

## 1. Introduction

In an era where technological advancements are profoundly reshaping various sectors, face recognition has emerged as one of the most impactful applications of artificial intelligence [1]. The development of systems capable of recognizing multiple faces simultaneously has become increasingly important across a wide range of real-world contexts, particularly within Internet of Things (IoT) environments. In smart home settings, such systems can enhance surveillance capabilities by integrating directly with IoT sensor networks [2]. Within healthcare, accurate patient identification supports secure access to medical services and facilitates continuity of care [3]. In the education sector, multi-face recognition can assist with automated attendance tracking and access control, while also enabling data-driven analysis of student participation trends and course engagement [4]. Furthermore, in the context of smart cities, multi-face recognition technologies contribute to public safety by supporting real-time monitoring and the identification of potential security threats during large-scale public events. These diverse application areas underscore the growing demand for scalable, efficient, and privacy-conscious face recognition systems tailored for decentralized deployment.

This growing need to distinguish multiple faces concurrently coincided with new efforts to develop a variety of facial recognition technologies. Based on these needs, developers have started to gather information on how a system could improve these procedures. Until now, CNN-based approaches (VGG16-19) have achieved SOTA(state-of-the-art) results based on precision [5].

However, just CNN-based approaches face several challenges when applied to real-world multi-face recognition tasks. First, they require large-scale training datasets and substantial computational resources. This makes both training and inference time-consuming, particularly in scenarios involving multiple subjects simultaneously. In the context of the Internet of Things (IoT), the need for computational power arises as a second challenge: IoT environments often involve constrained devices and decentralized data sources, making large-scale centralized computation impractical. But existing IoT-based facial recognition systems (e.g., [2,6]) still rely heavily on centralized servers or cloud infrastructures, which limits scalability and increases latency. Thirdly, CNN-based face recognition systems often handle only one face at a time.

In this paper, we propose a hybrid CNN–SVM system to overcome these challenges. Hybrid face recognition systems integrate two algorithms: one for feature extraction and another for classification. Prior studies have demonstrated that such designs not only shorten training time, but also lower overall computational costs [7]. Since CNNs, and especially VGG16, have consistently achieved state-of-the-art results in feature extraction, employing them in a hybrid architecture is a natural next step. In this work, we explore a hybrid system where VGG16 provides the feature representations and SVM performs the classification, aiming to combine accuracy with efficiency. While our system also requires computational resources, the burden is considerably lower when combining CNN feature extraction with a lightweight classifier such as Support Vector Machine (SVM). Unlike end-to-end CNN classification, training an SVM on extracted features is significantly less demanding, enabling faster model development and reduced computational overhead [8]. Our proposed hybrid approach is designed for decentralized deployment; it can run efficiently on small devices, such as IoT cameras or Raspberry Pi boards, without requiring centralized servers. Moreover, it supports the recognition of multiple faces simultaneously, an essential capability for real-time applications. Although hybrid CNN–SVM systems for single-face recognition have been studied [9], to the best of our knowledge, no prior work has extended this paradigm to hybrid multi-face recognition. Our system addresses this gap by leveraging the robustness of CNN-based feature extraction with the lightweight efficiency of SVM classification, thereby enabling accurate, real-time recognition of multiple individuals in resource-constrained IoT environments.

This study seeks to address the following overarching research question:

*How can a hybrid approach that combines CNN-based feature extraction with traditional machine learning classifiers improve the performance of face recognition systems compared to CNN-only models?*

To explore this question, this research involved constructing a custom dataset, evaluating different model architectures, and assessing the effectiveness of hybrid systems—specifically those combining deep feature extraction with classifiers such as Support Vector Machines (SVM). The investigation was further structured around the following four specific sub-questions:

- What are the most effective pre-processing techniques for robust multi-face recognition?
- How can CNN architectures for feature extraction be effectively combined with non-CNN classifiers to mitigate challenges such as overfitting, limited scalability, and parameter sensitivity in multi-face recognition tasks?
- How can state-of-the-art performance be achieved under the constraints posed by small datasets and edge deployment requirements?
- What architectural and implementation choices are necessary to realize a fully decentralized face recognition system suitable for IoT applications?

To answer these questions and systematically assess both existing and proposed systems, a set of evaluation criteria was defined. These criteria were developed based on limitations identified in the related work, and are as follows:

- *Small Dataset Robustness*: the system’s ability to maintain high performance with limited training data.
- *Multi-Face Recognition*: the capability to identify multiple individuals simultaneously in a single frame.
- *High Accuracy*: the ability to achieve recognition accuracy above 95%.
- *Hybrid Architecture*: use of a CNN for feature extraction (specifically VGG16) in conjunction with a non-neural classifier.
- *VGG16 Feature Extractor*: adoption of the VGG16 architecture for robust and transferable feature extraction.
- *SVM Classifier*: use of a Support Vector Machine in place of a CNN’s native classification layers, offering improved generalization on high-dimensional features.

These criteria were also applied to evaluate the proposed hybrid system in direct comparison with the approaches discussed in the Related Work section.

To validate the proposed system and assess its practical applicability, the following research steps were undertaken:

1. A hybrid face recognition system was developed by integrating CNN-based feature extraction (VGG16) with an SVM classifier, aiming to improve performance, reduce overfitting, and maintain efficiency on small datasets.
2. The system was evaluated against relevant state-of-the-art models, with performance comparisons conducted based on accuracy, runtime efficiency, and architectural suitability for decentralized inference.
3. Real-world applicability was assessed by testing the system under dynamic conditions using a diverse, custom-built dataset reflecting realistic environmental variability.

The proposed system contributes to the ongoing development of face recognition technologies by offering a practical and scalable solution tailored to resource-constrained environments, particularly IoT deployments. Applications span a variety of use cases, including security surveillance, access control, and organizational attendance tracking.

In particular, this study highlights the potential of hybrid models to outperform CNN-only systems in contexts where computational efficiency, ease of deployment, and small dataset robustness are critical. By decoupling feature extraction from classification, the architecture supports modular design, greater adaptability, and more efficient training pipelines—all of which are essential for future-proof edge AI deployments.

Thus, the system’s goal is to solve the drawbacks just CNN-based approaches [5], which were previously mentioned, being able to recognize multiple faces simultaneously and not just one face at a time [9], and, lastly and most importantly, offering a decentralized and secure system that is not prone to attacks or scalability issues [2].

Now that the idea of this paper has been outlined, the proceeding sections are organised as follows: Section 2 reviews similar publications and their lack of reaching the intended objectives. The suggested system design is presented in Section 3, together with information on the dataset, CNN-based feature extraction, SVM classification, workstation specifications, and camera. Section 4 describes the results of the system together with a differentiation of other state-of-the-art solutions. Section 5 discusses the results, limitations, and future opportunities. Finally, Section 6 concludes the paper and outlines directions for future research.

## 2. Related Work

Face recognition is a prominent discipline in computer vision and biometrics [10], and has been extensively researched for a variety of applications, including human–computer interaction and security systems [11]. Despite progress, challenges and limitations remain, including the following:

- Scalability: The ability to maintain efficacy as the size and complexity of a dataset increase.
- Efficiency: The ability to balance real-time constraints with computational demands.
- Durability: The ability to adapt to changes in environmental conditions, such as occlusions and illumination.

Hybrid approaches in multi-face recognition aim to combine the strengths of traditional methods with modern deep learning techniques to address issues like scalability, accuracy, and computational efficiency [12]. Historically, face recognition was dominated by methods such as principal component analysis and linear discriminant analysis because of their simplicity and ability to reduce dimensionality while conserving essential features. These methods established the foundation for face identification through statistical feature representations [9]. However, they could not meet the demands of dynamic real-world applications because they could not accommodate variations in pose, illumination, and expression. Deep learning’s ability to learn hierarchical and robust features directly from data has revolutionized face recognition. Convolutional Neural Networks (CNNs) have set new standards for facial recognition tasks, including AlexNet [13], VGGNet [14], and ResNet [15]. These architectures can extract intricate features, surpassing conventional methods in their ability to handle nonlinear variations, such as occlusion, pose, and expressions. Despite their success, CNNs are computationally expensive and often require substantial data to prevent overfitting. Hybrid systems balance computational efficiency and accuracy by using traditional classifiers for recognition and CNNs for feature extraction [16]. To extract facial features, these systems use pre-trained CNN models, such as ResNet or VGG16. These models are then combined with classifiers, such as Support Vector Machines (SVM) or K-Nearest Neighbors (KNN), for classification. For example, a face recognition system that uses CNN for feature extraction has been reported to achieve state-of-the-art results [17]. Hybrid multi-face recognition systems are a significant advancement because they combine the adaptability of deep learning with the efficacy of traditional methods. Further integration of optimization techniques could improve these systems’ effectiveness and practicality in real-world scenarios.

Since datasets substantially impacted the development, training, and evaluation of this multi-face recognition system, a detailed representation of the datasets used in the research is necessary. Focusing on their characteristics and importance, let us examine in detail the most frequently used ones. The LFW dataset, for example, is a benchmark dataset compiled from the web. It comprises 13,000 labeled face images of 5749 individuals. It is often used to evaluate algorithms for face verification and identification. It is well-suited for assessing the robustness of multi-face recognition systems due to variations in pose, illumination, and occlusion, as reported in [12,18]. The CASIA-WebFace dataset comprises over 494,000 images of 10,575 subjects. It is primarily used to train deep learning models for face recognition. It contains a diverse array of identities and expressions. The dataset is used for a hybrid protection face recognition system [19]. The VGGFace2 dataset comprises more than 3.3 million images of 9131 individuals, and focuses on variability in pose, age, and illumination. This approach is frequently implemented in hybrid recognition systems to train deep models, including ResNet 50 [20].

Several approaches were combined to improve the accuracy and resilience of hybrid face recognition systems. Using Convolutional Neural Networks (CNNs) for feature extraction in combination with traditional classifiers, such as Support Vector Machine (SVM), can enhance recognition accuracy. This method utilizes SVM’s efficient classification and CNN’s deep feature extraction capabilities [21]. After completing the general literature review, we examined the extent to which existing publications met the specific criteria established for this study. These evaluation criteria are detailed in Table 1, which provides a comparative overview of the related work.

Accuracy remains a central benchmark in face recognition research. While most existing approaches report competitive accuracy, an important exception is the foundational work by Turk and Pentland [29], which, although not included in Table 1, remains influential for introducing principal component analysis (PCA) into face recognition. Insights from this work informed our cross-validation procedures and the tuning of our model parameters.

A review of the literature reveals that several studies offer partial solutions. For instance, a comparative analysis of convolutional architectures, including VGG16, VGG19, and various ResNet variants, was conducted in [5]. VGG16 demonstrated the most favorable performance in this analysis. Then, a hybrid approach that paired VGG16 with an SVM classifier was proposed in [5]; however, their implementation was limited to recognizing one face at a time. Other studies relied on deep architectures for face recognition: a ResNet50 was used in [20], while a hybrid model integrating a time series prediction module with an SVM was exploited in [19]. Neither of them addressed multi-face recognition explicitly.

Several recent studies have explored deep learning architectures for face recognition in various contexts. For example, Mittal et al. [12] employed ResNet34 in a multi-face recognition setting; however, their focus was not on hybrid systems or decentralized deployment. Similarly, Kavita et al. [18] conducted comparative evaluations using the FER and LFW datasets, but their work did not address simultaneous multi-face recognition.

Other developments investigated hybrid CNN–SVM models, although often with different objectives. For instance, Basly et al. [16] proposed a ResNet-based hybrid model tailored for human activity recognition, while Schroff et al. [23] achieved state-of-the-art performance on the YouTube Faces dataset using FaceNet embeddings, targeting single-face identification rather than multi-face scenarios.

The feasibility of large-scale image classification using a Convolutional Neural Network (CNN) trained on ImageNet was demonstrated in early work by Krizhevsky et al. [13], which laid the foundation for later architectures such as VGGNet [14]. VGG remains a strong baseline for feature extraction tasks due to its simplicity and transferability. Parkhi et al. [17] extended CNN architectures to incorporate age progression, while Mohammad et al. [2] implemented lightweight face recognition models—MobileNetV2 and FaceNet—on Raspberry Pi for IoT applications. Xie et al. [6] proposed an edge-based privacy-preserving recognition framework; however, their system did not support hybrid classification or multi-face scenarios.

Additional research has focused on lightweight feature extractor designs suitable for edge computing. EdgeFace and xEdgeFace [25,26] proposed optimized CNN-based models specifically for edge deployments. FaceLiVT [24] introduced a mobile-efficient architecture combining CNNs with Transformer blocks. These approaches integrate feature extraction and classification into a single pipeline, unlike our method, which decouples these stages to enhance modularity and adaptability across deployment scenarios.

Recent work has increasingly targeted face recognition on embedded IoT platforms. For example, MultiTask-Face [27] implemented a multi-attribute recognition model on Raspberry Pi, capable of predicting identity, age, and ethnicity. However, its reliance on a pure CNN architecture limited its robustness when trained on small datasets. Another approach combined MTCNN-based face detection with an edge–cloud hybrid framework to improve accuracy and throughput [28], but remained dependent on centralized infrastructure, thereby lacking a fully decentralized architecture.

In summary, no prior study has proposed a hybrid system integrating VGG16 for feature extraction and SVM for classification to perform decentralized, multi-face recognition on small datasets within IoT environments. While previous work has investigated hybridization or edge deployment independently, none fully align with the comprehensive set of evaluation criteria defined in this study.

The system presented here fills this gap by enabling simultaneous recognition of multiple faces under constrained conditions without relying on cloud-based infrastructure. Its architecture is especially well suited for applications such as surveillance, access control, and smart building management, where privacy, speed, and decentralized processing are critical requirements.

## 3. Materials and Methods

**Figure 1:**
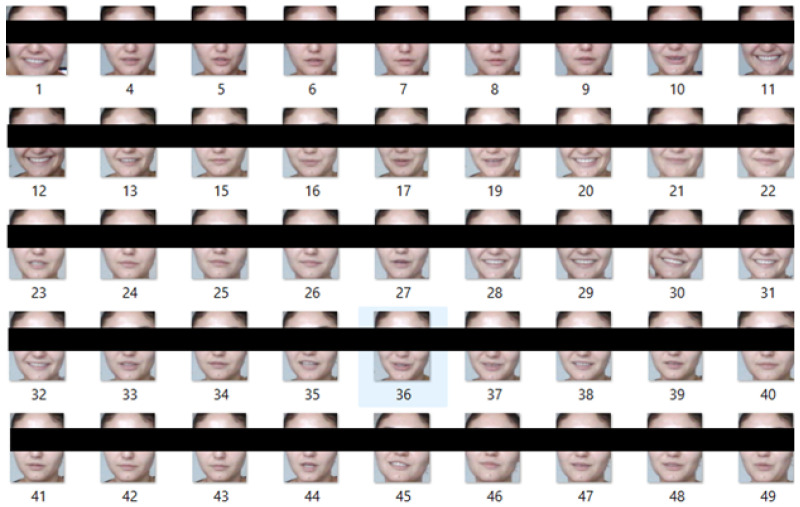
Snapshot of the picture collected for the dataset.

Before detailing the data collection process, it is important to clarify the requirements that guided the dataset design. Specifically, the dataset was constructed to meet the following criteria: (i) limited size to simulate small-scale training conditions common in IoT deployments; (ii) diversity in facial expressions to enhance model robustness; (iii) variation in lighting conditions, reflecting real-world environments; and (iv) acquisition under realistic, unconstrained settings. These characteristics were intentionally selected to align with the practical constraints of decentralized, camera-based face recognition systems operating in IoT contexts. In this regard, the dataset was tailored to ensure compatibility with resource-limited environments while maintaining support for accurate multi-face identification. An overview of the collection of pictures of our dataset is presented in Figure 1; the following section provides a detailed account of the data collection methodology.

**Figure 2:**
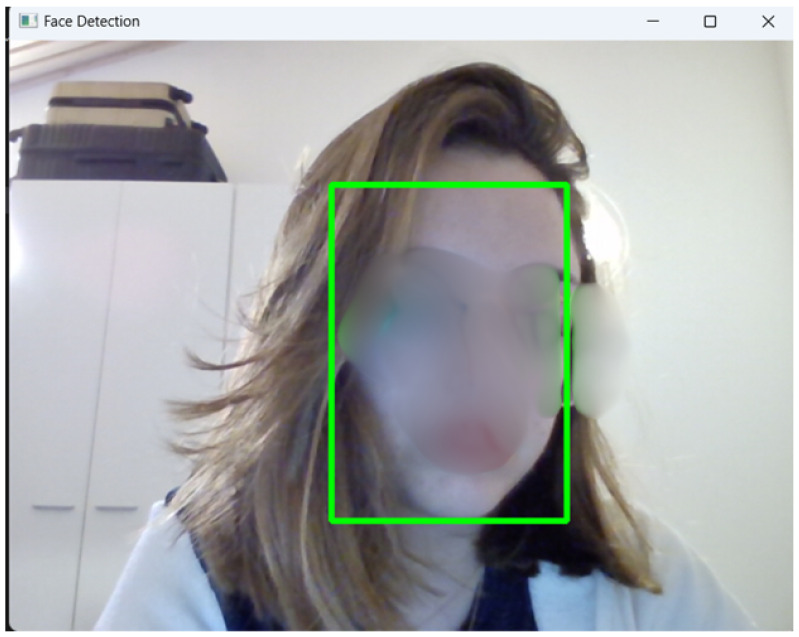
Example of the dataset collection process using the MTCNN algorithm. The green box highlights the identification made by MTCNN algorithm.

The dataset was collected using voluntary contributions from friends and family members. Images of each subject were captured via a webcam using a custom acquisition script that integrates the Multi-task Cascaded Convolutional Neural Network (MTCNN) algorithm. An example of the acquisition process is shown in Figure 2. This algorithm was selected due to its high accuracy and proven effectiveness in face-detection tasks, as reported in recent comparative evaluations [30]. The MTCNN pipeline performs face detection, facial landmark localization, and bounding box regression simultaneously, making it particularly suitable for applications that require the precise identification of facial feature points, such as the eyes, nose, and mouth. These capabilities were essential for ensuring consistency and quality in the cropped face images used for training and evaluation. Moreover, the MTCNN implementation integrates seamlessly with the OpenCV library, which was employed in this work to support real-time detection and image preprocessing.

All participants provided informed consent prior to data collection, and the images were anonymized and used exclusively for the purpose of academic research. No biometric or sensitive personal information was stored beyond facial imagery, and all data handling was conducted in compliance with ethical research standards.

**Figure 3:**
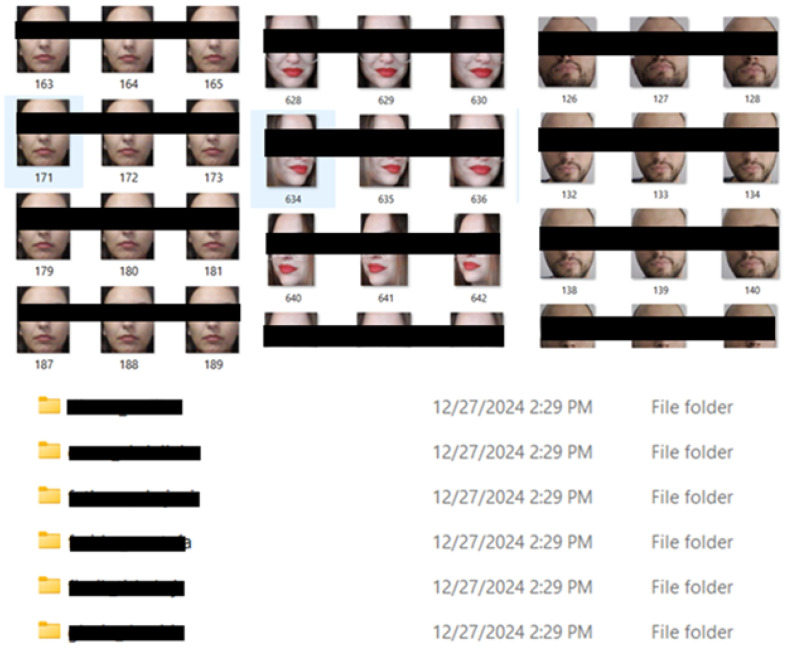
Process of dataset collection.

Each subject in the dataset is represented by a dedicated folder containing approximately 2100 images, acquired under varying lighting conditions, facial expressions, and head poses. In total, the dataset consists of 20 folders—corresponding to 20 individuals—yielding a combined total of 42,000 face images. This comprehensive dataset was divided into three subsets: 1500 images per subject for training, 300 for validation, and 300 for testing. This stratified split was designed to ensure a balanced and diverse distribution of data across different stages of the modeling pipeline. A summary of the dataset composition is provided in Table 2, while Figure 3 offers a visual representation of the dataset structure to aid in understanding.

With the dataset preparation complete, the feature extraction and classification components of the system are described below.

### 3.1. Feature Extraction Using VGG16

**Figure 4:**
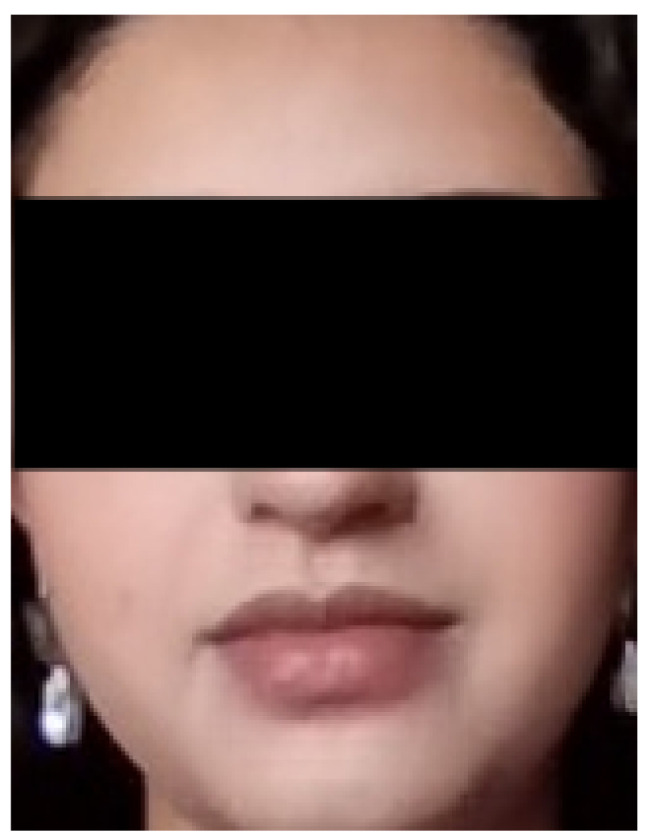
Input image.

**Figure 5:**
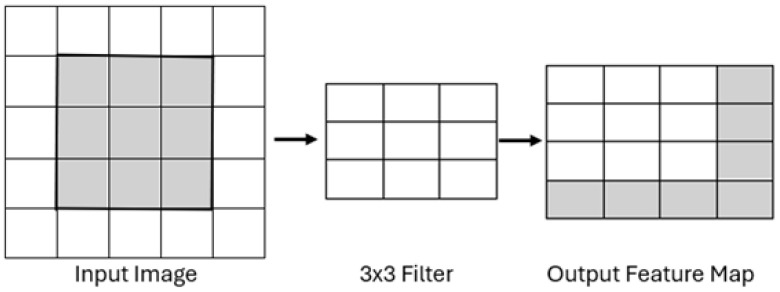
Convolution layers procedure.

**Figure 6:**
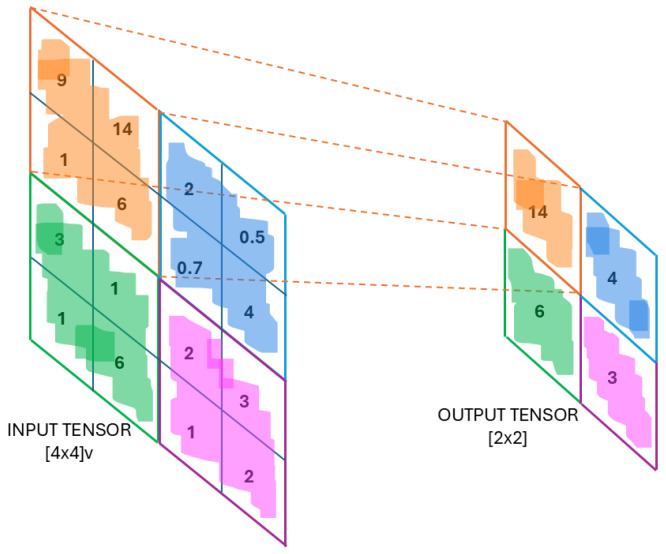
Max pooling operation [31].

**Figure 7:**
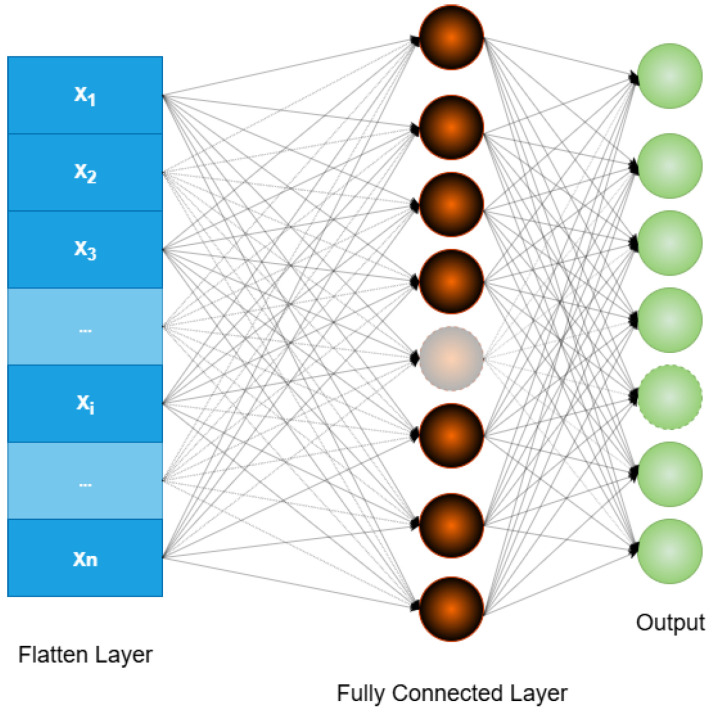
Connected layers.

The VGG16 architecture was employed as the feature extractor due to its proven effectiveness in computer vision tasks. The network is composed of several key stages:

1. *Input layer:* The model processes input RGB images of size 224×224×3; an example is shown in Figure 4.
2. *Convolutional Layers:* These layers apply a series of 3×3 filters to capture low- to high-level features, such as edges, textures, and shapes. Each convolutional layer uses a ReLU activation function, and the number of filters increases progressively (e.g., 64, 128, 256, 512) as the network depth increases, thereby allowing for the model to learn more abstract representations. This process is illustrated in Figure 5.
3. *Pooling Layers:* Max pooling operations follow selected convolutional blocks to reduce spatial dimensions while preserving the most salient information. This downsampling mechanism improves computational efficiency and reduces overfitting by retaining dominant features only. Figure 6 depicts the pooling process.
4. *Fully Connected Layers:* The high-level features extracted by the convolutional layers are flattened and passed through fully connected layers to create a dense feature representation. As shown in Figure 7, these layers connect each neuron to every unit in the previous layer, producing a discriminative vector embedding of the input image.
5. *Softmax Output Layer:* In the original VGG16 architecture, the final layer applies a softmax activation function to classify input images. However, in this study, the softmax layer was excluded. Instead, the output from the last max pooling layer (post-flattening) was used as a 25,088-dimensional feature vector, which served as input to a Support Vector Machine (SVM) classifier.

### 3.2. Classification with SVM

The classification module uses a linear SVM with a penalty parameter C=10, selected through a grid search to achieve a balance between classification accuracy and generalization. The SVM was chosen over alternative classifiers, such as K-Nearest Neighbors (KNN) and softmax-based neural classifiers, due to its superior performance in high-dimensional feature spaces and its efficiency on resource-constrained IoT hardware. The SVM’s robustness to small datasets and its well-established theoretical foundation further supported this decision.

Although SVMs are inherently binary classifiers, the system leverages the One-vs-One (OvO) strategy from the scikit-learn library to support multi-class classification. For *K* classes, OvO constructs $\frac{K(K-1)}{2}$ binary classifiers, each trained to distinguish between a unique pair of classes. Final predictions are made through a majority voting scheme across all binary classifiers, with the class receiving the highest number of votes being selected as the predicted label.

To improve reliability in real-world scenarios, a confidence threshold was applied to the SVM output. Predictions falling below this threshold were labeled as “Unknown”, thereby enabling the system to reject low-confidence classifications and reduce false positives in open-set recognition settings.

**Figure 8:**
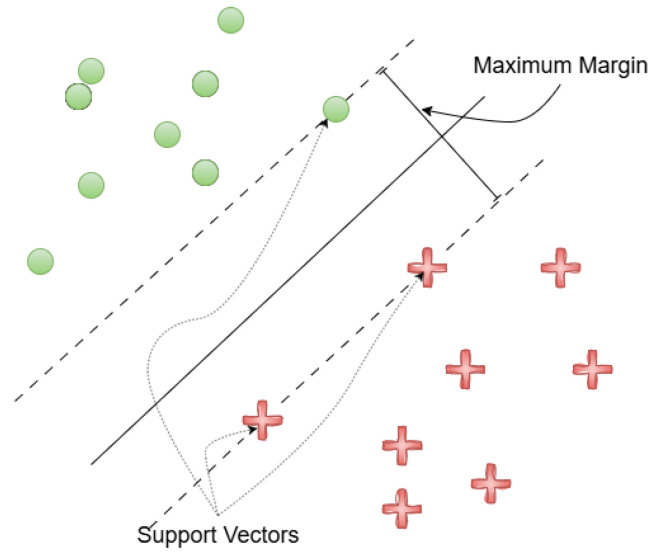
Classification with SVM. The SVM identifies a separator with a maximum margin that separates the red crosses from the green points.

A conceptual overview of SVM decision boundaries is presented in Figure 8, which illustrates how the algorithm identifies an optimal hyperplane to separate classes while maximizing the margin between them.

**Figure 9:**
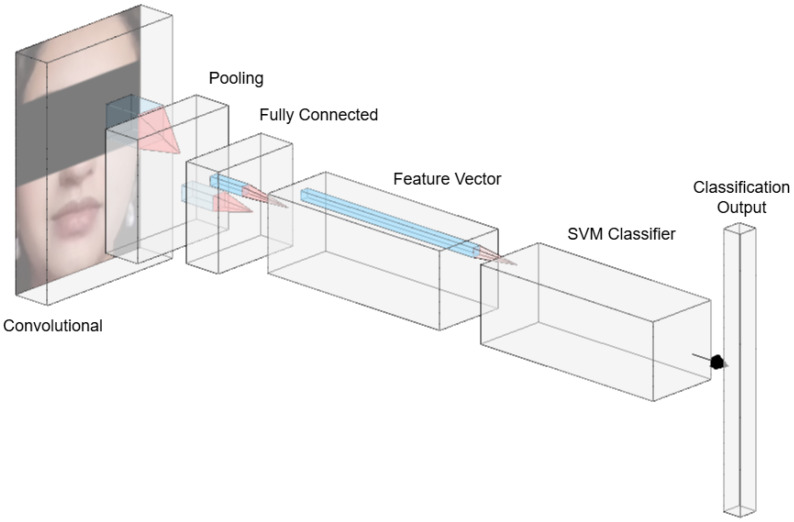
Overview representation of the hybrid architecture.

Figure 9 gives a schematic overview of the hybrid system.

**Figure 10:**
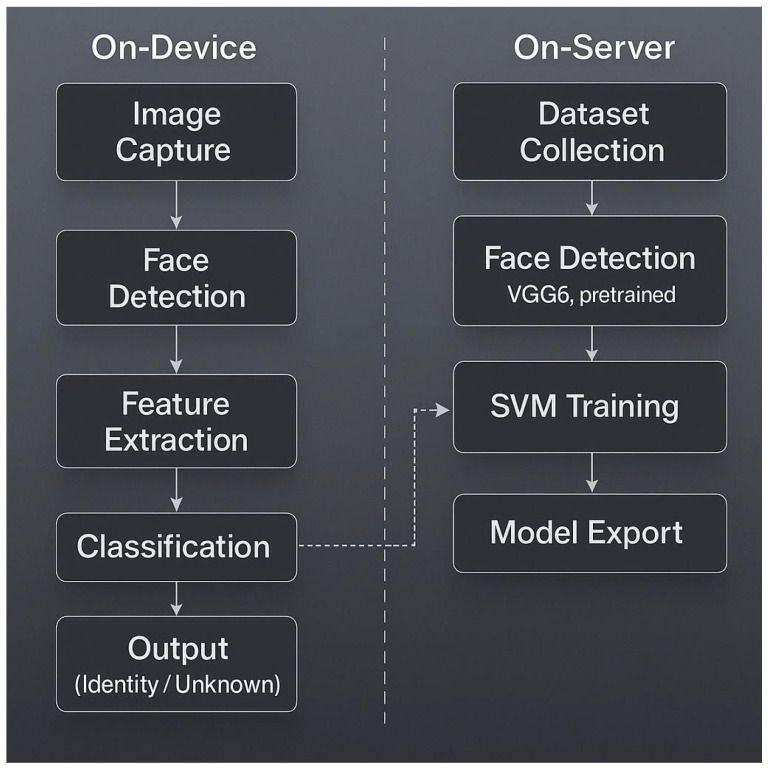
Overview of the proposed hybrid multi-face recognition pipeline. The system is divided into on-server (training) and on-device (inference) components. VGG16 is used for feature extraction, and an SVM classifier is trained on extracted features and exported to the device for real-time, decentralized classification. The dashed arrows indicate the deployment of the trained model from the server to the IoT device for local inference.

In Figure 10, an overview of the proposed hybrid multi-face recognition pipeline is shown. The architecture is organized into two main components: an on-server (training) environment and an on-device (inference) environment. During training, VGG16 is employed for deep feature extraction, and the extracted features are used to train a Support Vector Machine (SVM) classifier. The resulting trained model is then exported to the target IoT device for decentralized, real-time inference. Dashed arrows in the figure indicate the deployment process from the centralized training server to the distributed edge device.

The experiments were conducted using two types of hardware: a high-performance workstation for training, and a resource-constrained IoT camera for real-time inference. The specifications of the workstation are provided in Table 3, while Table 4 details the hardware characteristics of the IoT device.

The selected IoT camera addressed several limitations associated with conventional laptop webcams, including restricted field of view and limited multi-face coverage. Its integration into the system enabled accurate detection and classification of multiple faces in real-world conditions, further validating the feasibility of decentralized inference.

The implementation of the proposed system relied on a range of open-source libraries and tools. The Scikit-learn library was used for SVM training and performance evaluation, while OpenCV supported real-time face detection and image processing. TensorFlow 2.18.0 served as the primary framework for developing and training the VGG16-based feature extractor. Additional libraries such as Joblib were utilized for efficient serialization of trained models, and NumPy facilitated numerical computations and dataset manipulation. Together, these tools enabled a streamlined pipeline encompassing data preparation, model training, deployment, and inference.

In addition to the hardware components, a robust and maintainable software infrastructure was critical for the development and reproducibility of the system. Python 3.10 was selected as the main programming language due to its strong support for machine learning and deep learning frameworks. Visual Studio Code (version 1.100) was used as the integrated development environment (IDE), offering effective features for code management, debugging, and version control.

A complete list of software libraries and tools used in this work is presented in Table 5.

## 4. Results

The primary objective of this work was to develop a decentralized multi-face recognition system suitable for real-time deployment on resource-constrained Internet of Things (IoT) devices. The system was designed to (i) recognize multiple faces simultaneously, (ii) distinguish between enrolled and non-enrolled individuals, and (iii) achieve competitive accuracy while operating entirely on-device.

**Figure 11:**
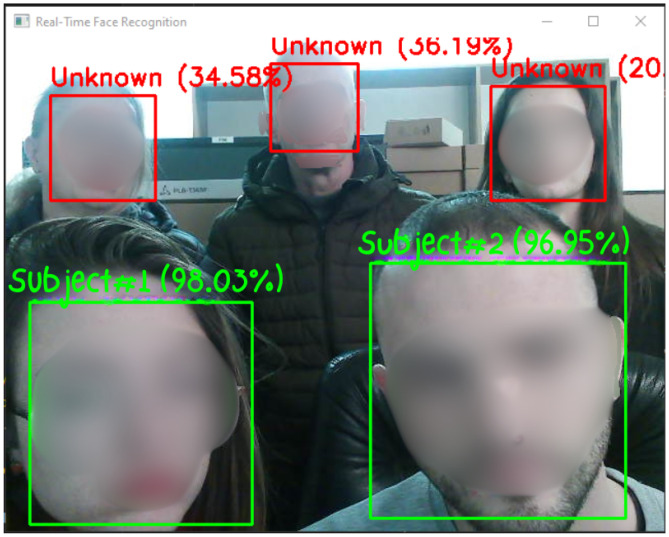
Overviewof subject recognition results across the dataset. The system accurately classifies the majority of individuals providing the probability for each subject.

To evaluate the system’s performance, we conducted experiments using a custom dataset composed of 20 subjects, with each subject represented by 2100 facial images captured under varying lighting conditions and expressions. As illustrated in Figure 11, the system successfully identified the majority of individuals with high accuracy.

**Figure 12:**
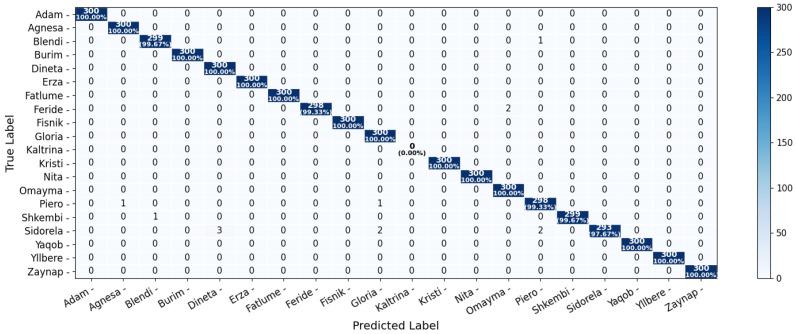
Confusion matrix showing classification performance per subject. The matrix helps visualize class-wise accuracy and potential confusion between subjects.

A quantitative breakdown of performance is shown in Figure 12, which presents the confusion matrix summarizing classification outcomes per subject. Recognition rates exceeded 99% for most individuals, with several achieving perfect classification (100%). However, the system failed to correctly classify any images for Subject #11, resulting in a recall and F1-score of 0.00. This failure is attributed to motion blur and inconsistent image capture during data collection for this subject, which likely disrupted the consistency of feature vectors and led to systematic misclassification. In effect, none of the true samples were correctly retrieved (TP = 0), leading to zero recall and, by extension, a zero F1-score.

Despite this isolated failure, the overall system performance remained strong, achieving an average classification accuracy above 95%. Furthermore, the model correctly labeled previously unseen individuals as “Unknown”, validating its capability to reject non-enrolled subjects and supporting its use in open-set, real-time environments.

To contextualize these results, we compared our method with recent state-of-the-art systems. For instance, the multi-task face recognition model on Raspberry Pi proposed in [27] achieved approximately 99% accuracy, but focused primarily on attribute prediction tasks (identity, age, ethnicity) and relied on a CNN-based softmax classifier. Similarly, the edge–cloud hybrid framework in [28] improved frame rate via cloud offloading, but sacrificed decentralization and introduced potential privacy vulnerabilities due to biometric data transmission over networks.

In contrast, the proposed VGG16 + SVM hybrid architecture achieves high accuracy while operating entirely on-device. By avoiding cloud reliance, the system significantly reduces the exposure of sensitive facial data to external servers, thus mitigating risks of interception or unauthorized access. Additionally, the inclusion of an *Unknown* class enhances robustness by preventing the misclassification of non-enrolled individuals and reducing the risk of impersonation or erroneous enrollment.

These findings confirm the feasibility and effectiveness of employing a lightweight hybrid architecture for scalable, decentralized, and privacy-preserving multi-face recognition in IoT applications.

## 5. Discussion

This study demonstrates the practical feasibility of a decentralized, hybrid multi-face recognition system tailored for Internet of Things (IoT) environments. The proposed framework addresses key limitations associated with traditional Convolutional Neural Network (CNN)-based solutions, including their dependence on centralized computational infrastructure, high processing demands, and limited scalability for real-time applications.

By combining VGG16 as a pre-trained deep feature extractor with a lightweight Support Vector Machine (SVM) classifier, the system achieves a favorable balance between computational efficiency and recognition accuracy. This design makes it particularly suitable for embedded platforms such as IoT cameras and edge devices, where processing power, memory, and energy resources are constrained.

In contrast to prior works that focus primarily on single-face recognition or rely on cloud-based processing, the proposed system is capable of recognizing multiple individuals simultaneously at the edge. The achieved accuracy of over 95% on a custom dataset—with substantial variation in lighting conditions and facial expressions—highlights the model’s robustness and its suitability for deployment in real-world, unconstrained scenarios.

Notably, the system exhibited a performance failure in the case of Subject #11. This outlier reflects the model’s sensitivity to inconsistencies in data quality, likely caused by motion blur and the subject’s inability to remain stationary during image acquisition. This observation reinforces the importance of high-quality data collection practices, including consistent labeling, stable capture conditions, and the potential utility of data augmentation, denoising techniques, and advanced facial alignment algorithms to improve overall reliability.

Looking forward, several research directions are envisioned. These include integrating more efficient CNN backbones such as MobileNet or EfficientNet, as well as exploring transformer-based architectures for feature extraction. Additionally, evaluating the model on large-scale public datasets will help assess its generalizability beyond the current use case. Privacy and security aspects may be further strengthened by incorporating federated learning techniques or blockchain-based identity management frameworks, ensuring that decentralized inference remains both trustworthy and resilient.

## 6. Conclusions

This study introduces a lightweight, decentralized, hybrid multi-face recognition system specifically designed for IoT applications. By integrating the VGG16 architecture for deep feature extraction with a Support Vector Machine (SVM) classifier, the system achieves both high accuracy and computational efficiency on resource-limited devices. The model reaches an average inference speed of 4.16 FPS ± 1.57, which falls within an acceptable range for IoT-based face recognition applications. The proposed framework addresses several core challenges in the domain, including real-time multi-face detection, reduced computational overhead, and the elimination of reliance on centralized processing infrastructure.

Empirical evaluation on a custom dataset of 20 subjects demonstrated the system’s robustness under varied conditions, achieving classification accuracy exceeding 95%. While the current implementation is based on VGG16, future work will explore more compact and efficient architectures, such as MobileNet and Transformer-based backbones, to further optimize performance. Additionally, expanding the dataset and evaluating the system on standardized public benchmarks will help assess its generalizability. To further strengthen privacy and security, the integration of federated learning and privacy-preserving mechanisms is also envisioned.

Rather than pursuing a comprehensive architectural benchmarking of face recognition models, this study focused on demonstrating the feasibility and effectiveness of a lightweight hybrid solution for decentralized multi-face recognition in IoT scenarios. Unlike state-of-the-art systems such as ArcFace, CosFace, and FaceNet—which are typically designed for cloud or server-based environments and trained on large-scale datasets—our system targets embedded platforms characterized by limited memory, power, and latency constraints.

While it would be possible to benchmark against other configurations such as standalone VGG16 classifiers, ResNet combined with an SVM, or MobileNetV2-based pipelines, aligning training regimes, optimization settings, and runtime conditions across architectures would deviate from the practical constraints imposed by our deployment context. In line with application-driven research strategies, such as that adopted in SPECTRE [32], we prioritized validating key functional requirements—namely, real-time multi-face recognition on IoT devices, privacy-preserving on-device processing, and decentralized system architecture—over exhaustive architectural comparisons. This focus ensures the relevance and deployability of the proposed system in realistic IoT settings.

## Figures and Tables

**Table 1:** Comparative summary of existing face-recognition systems based on six evaluation criteria: dataset size suitability, capability for multi-face recognition, classification accuracy, hybrid model usage, utilization of VGG16 for feature extraction, and use of SVM as a classifier. The proposed system uniquely satisfies all criteria.

Publication	Small Dataset	Multi-Face Recognition	High Accuracy	Hybrid	VGG for Feature Extraction	SVM for Classifier
[2]	✓	✗	✓	✓	✗	✗
[5]	✗	✓	✓	✗	✓	✗
[6]	✓	✗	✗	✗	✗	✗
[7]	✗	✗	✓	✓	✓	✓
[12]	✗	✓	✓	✓	✗	✗
[13]	✗	✗	✓	✗	✗	✗
[14]	✗	✗	✓	✗	✓	✗
[16]	✗	✗	✓	✓	✗	✓
[17]	✗	✓	✓	✗	✓	✗
[18]	✓	✗	✓	✗	✗	✗
[19]	✗	✗	✓	✓	✗	✗
[22]	✗	✗	✓	✓	✗	✓
[23]	✗	✗	✓	✗	✗	✗
[24]	✗	✗	✓	✓	✗	✗
[25]	✗	✗	✓	✓	✗	✗
[26]	✗	✗	✓	✓	✗	✗
[27]	✓	✓	✓	✗	✗	✗
[28]	✗	✓	✓	✗	✗	✗
Proposed System	✓	✓	✓	✓	✓	✓

**Table 2:** Dataset split per subject and totals.

Subset	Per Subject	Total (20)	Percent	Grand Total
Training	1500	30,000	70%	
Validation	300	6000	15%	
Testing	300	6000	15%	
Total	2100	42,000	100%	42,000

**Table 3:** Workstation specifications used for experiments.

Component	Specification
Operating System	Windows 10 Pro 64-bit (Build 19045), Redmond, WA, USA
System Model	HP Z8 G5 Workstation Desktop PC
BIOS Version	U60 Ver. 01.02.07
Processor	Intel^®^ Xeon^®^ Gold 5418Y (48 CPUs), 2.0 GHz, Santa Clara, CA, USA
Memory (RAM)	32,768 MB RAM
Page File Size	7939 MB used, 34,705 MB available
DirectX Version	DirectX 12

**Table 4:** Specifications of the webcam used for real-time recognition.

Component	Specification
Model Name	Redragon HITMAN GW800
Category	USB Streaming Webcam
Power Rating	5 V, 250 mA
Serial Number	GW8002010165220
Producer	Red Digital Cinema, 94 Icon Foothill Ranch, CA, USA

**Table 5:** Specifications of the libraries.

Library/Tool	Version
TensorFlow	2.18.0
NumPy	2.0.2
OS	nt
joblib	1.4.2
OpenCV	4.10.0

## Data Availability

Data are available upon a reasonable request to the corresponding author.

## Abbreviations

The following abbreviations are used in this manuscript:

SOTA	State of the Art
SVM	Support Vector Machines
SVC	Support Vector Classifier
CNN	Convolutional Neural Networks
IoT	Internet of Things
